# Synchronous mucinous metaplasia and neoplasia of the female genital tract with both pulmonary metastases and STK11/KRAS gene mutations: a case report

**DOI:** 10.3389/fonc.2023.1246821

**Published:** 2023-11-13

**Authors:** Ru Wang, Hao Yu, Ming Liu, Ting Hao, Xiangyu Wang, Lianbao Cao

**Affiliations:** Shandong Cancer Hospital and Institute, Shandong First Medical University and Shandong Academy of Medical Sciences, Jinan, China

**Keywords:** SMMN-FGT, STK11, KRAS, pulmonary metastases, gene mutations

## Abstract

Multiple morphological changes in two or more sites of concurrent multifocal mucinous lesions in the female genital tract are indicative of SMMN-FGT, which is unrelated to high-risk HPV infection. MUC6 and HIK-1083 showed positive characteristic immunohistochemistry. Seldom is the condition described. Here we describe an SMMN-FGT patient who also had lung metastases and STK11/KRAS gene mutations. Based on the current researches, we hypothesize that SMMN-FGT is closely associated with the development of cervical gastric adenocarcinoma.

## Introduction

First reported and described by Mikami in 2009 (1), synchronous mucinous metaplasia and neoplasia of the female genital tract (SMMN-FGT) is characterized by several morphological changes in two or more sites of concurrent multifocal mucinous lesions in the female genital tract, including non-neoplastic, benign, junctional, and malignant features. Seldom is the condition described, and there is no consensus regarding its frequency, clinical presentation, pathological characteristics, diagnosis, treatment, or prognosis. In this case report, we describe an SMMN-FGT patient who also had lung metastases and STK11/KRAS gene mutations.

## Case report

**Figure 1:**
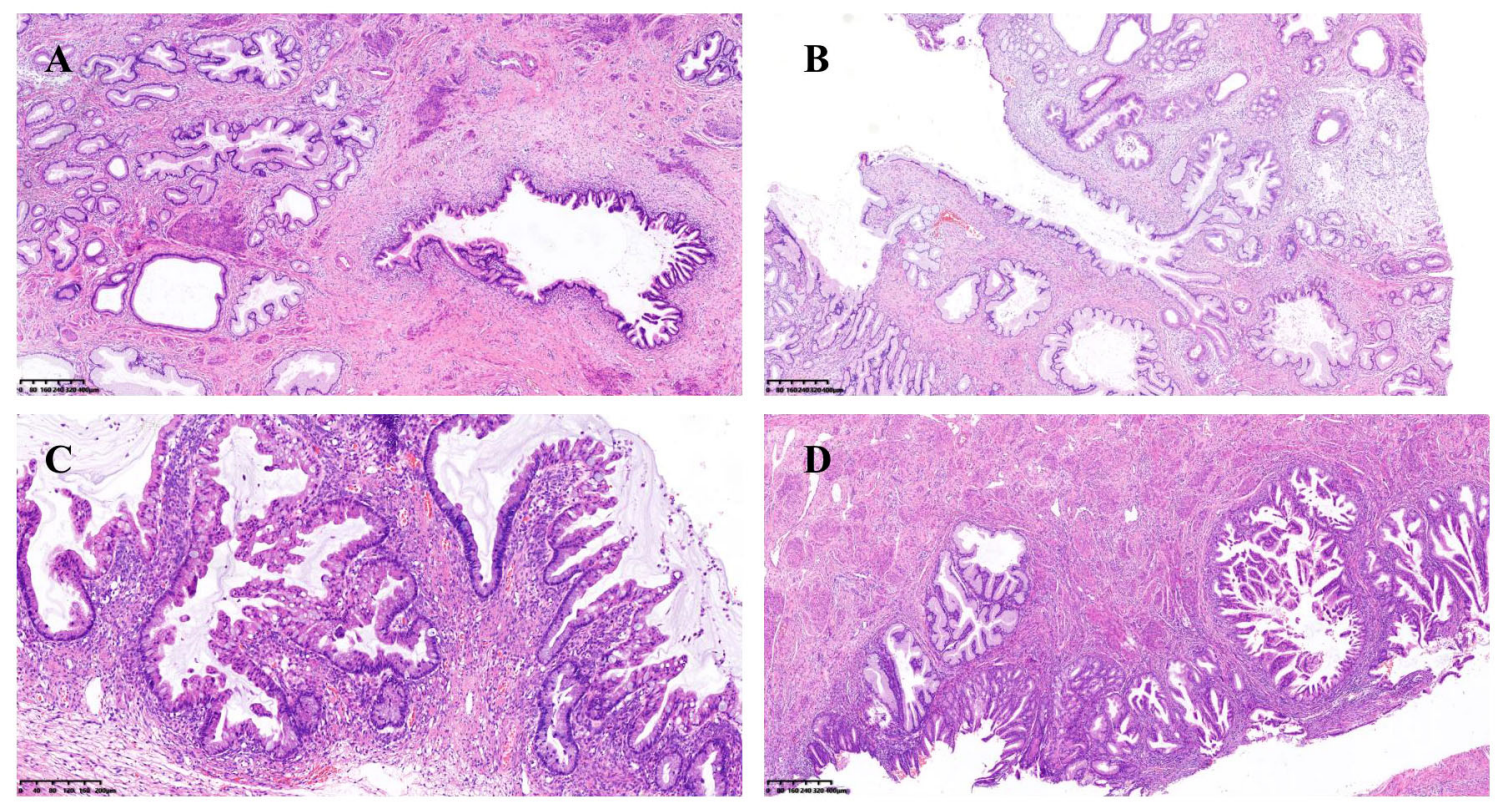
**(A)** Lobular glandular hyperplasia and atypical epithelial hyperplasia of the uterine cervix; **(B)** Lobular glandular hyperplasia and adenocarcinoma in situ of the uterine cervix; **(C)** Junctional mucinous adenoma of the ovary; **(D)** Mucinous epithelial metaplasia and mucinous adenocarcinoma of the endometrium.

During medical examination, a 45-year-old woman was brought to the nearby hospital where it was discovered that she had an “adnexal cyst” in her unit. The human papillomavirus test (HPV) showed HPV subtype negative. She had already received two doses of the vaccination with a 2-month gap before the medical examination and had never developed Covid-19/Omicron infection. Test results for the patient’s serum tumor markers of Cancer antigen125 and Cancer antigen19-9 were within limits. Her pelvic MRI was performed by medical professionals at the neighborhood hospital, which identified a cystic lesion in the right adnexal region and thickened endometrium. Several tiny nodular lesions were seen in both lungs during a chest CT scan. Her pathology revealed that she had a highly differentiated mucinous adenocarcinoma after the endometrial curettage operation. Then the bilateral salpingo-oophorectomy, bilateral omentectomy, pelvic lymph node and para-aortic lymph node dissection, and pelvic adhesion release were all carried out in addition to the whole abdominal uncomplicated hysterectomy. The endometrium’s mucinous adenocarcinoma (cervical type) was highly differentiated and penetrated the myometrium to a depth of about one-third of the uterine wall. There was no obvious intravascular cancer thrombus. The right ovarian tumor had an increased volume of around 13cm*10cm*10cm and two mucinous cystadenoma and junctional mucinous neoplasm components. Lobular glandular hyperplasia, atypical epithelial hyperplasia and adenocarcinoma *in situ* of the uterine cervix (**Figure 1**). Both the fallopian tubes and the left ovary were healthy. The tissue of the vaginal wall remained unaffected by the tumor, and there was no evidence of malignant tissue metastasizing into the pelvic and para-aortic lymph nodes.

**Figure 2:**
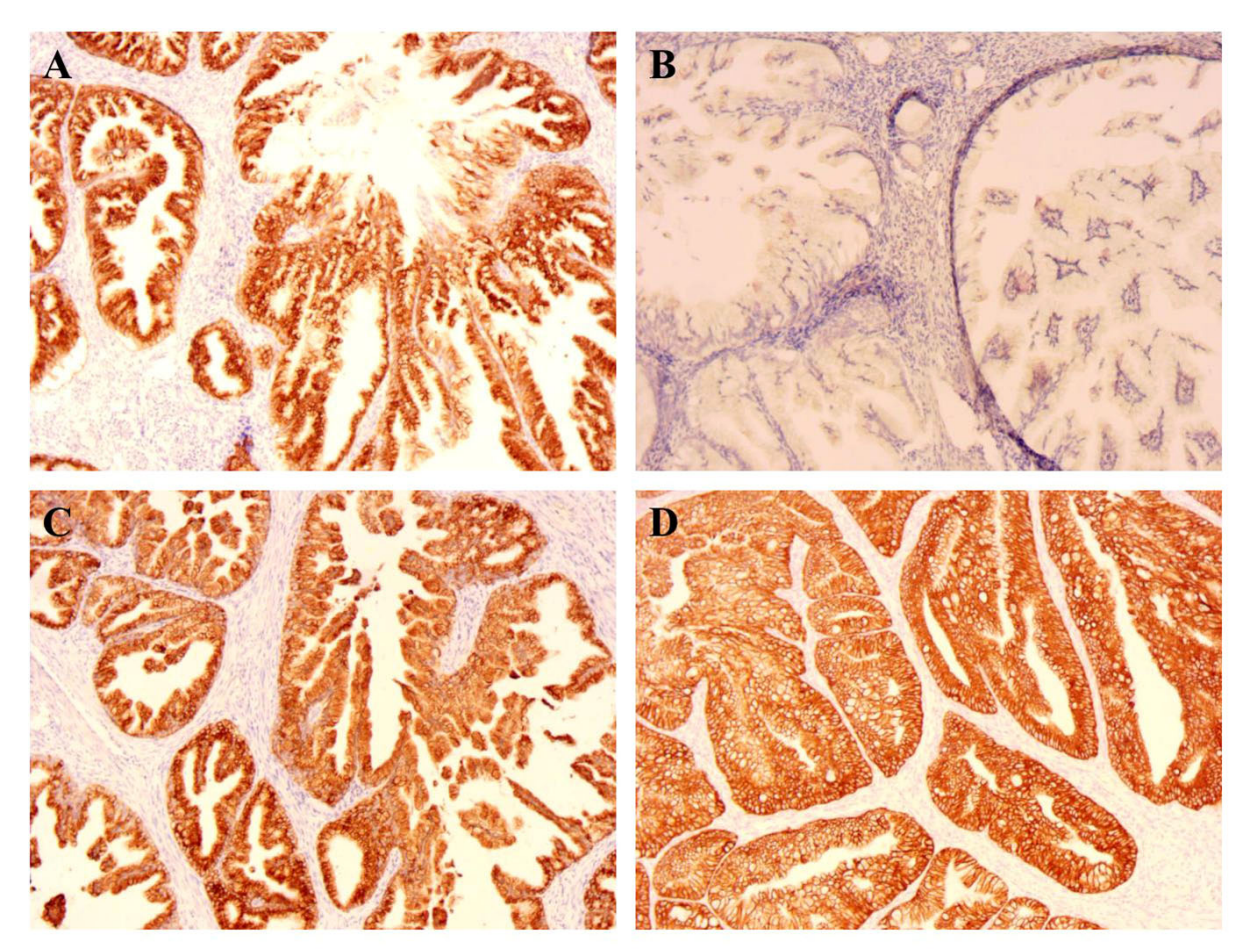
IHC of endometrium (×100). **(A)** The MUC6 expression of endometrium; **(B)** The MUC2 expression of endometrium; **(C)** The MUC5ac expression of endometrium; **(D)** The CK7 expression of endometrium.

Using immunohistochemistry (IHC), tumors including endometrium and the mucinous carcinoma of the right ovarian showed a strikingly similar pattern; both were positive for MUC6 and MUC5AC (**Figure 2**). The ER, PR, and p16 were negative for endometrium mucinous adenocarcinoma (**Table 1**). The ki-67 expression index of cervical tumor was about 30%, whereas the index of endometrial tumors was between 10% and 20%.

**Table 1:** Immunohistochemistry of different lesion sites.

	Postoperative diagnosis	MUC6	MUC2	MUC5AC	ER	PR	P53	CK7	CK20	COX-2	P16	Pax-8	CDX2-88
Cervix	adenocarcinoma in situ	–	–	+	–	–					–		
Endometrium	mucinous adenocarcinoma	+++	+	+++	–	–	–	+	–	–	–	+	–
Right ovary	mucinous cystadenoma and junctional mucinous neoplasm components	+++	+	+++									
Lung	mucinous adenocarcinoma						+	–				–	–

After surgery, the patient didn’t receive adjuvant treatment. A second CT at 7 months after surgery revealed metastases in both lungs. The patient then visited our hospital for additional testing. We gave her a PET/CT scan, which revealed many nodular tumors in both lungs, with possible metastases. The patient was then treated with liposomal paclitaxel and carboplatin, a first-line chemotherapy strategy for recurrent endometrial cancer. The larger lung lesion, which measured about 1.3 cm in diameter and was positioned in the lower right lobe’s posterior portion on a repeat CT, had dramatically advanced after two cycles of treatment. A CT-guided puncture biopsy of the right pulmonary nodule was performed, and the pathology showed mucinous adenocarcinoma was detected. The right pulmonary nodule tumor had the following IHC markers: CK7 (+), TTF-1 (-), CK20 (-), Villin (-), CDX2 (-), SATB2 (-), PAX-8 (-), and NapsinA (-). We conducted a genetic analysis on the patient’s endometrial mucinous adenocarcinoma tumor tissue and found a total of six genetic variations. KRAS missense mutations, such as the exon 4 missense mutation and STK 11 nonsense mutations, are among these clinically important mutations (exon 1 nonsense mutation). Four more variants were found by us, including the FGA missense mutation, GRIN2A missense mutation, KMT2V shift mutation, and PTPRD missense mutation, but their clinical significance has not yet been determined. Tumour mutational burden (TMB): 2.35muts/Mb. Microsatellite Instability (MSI): Microsatellite stability (MSS).

**Figure 3:**
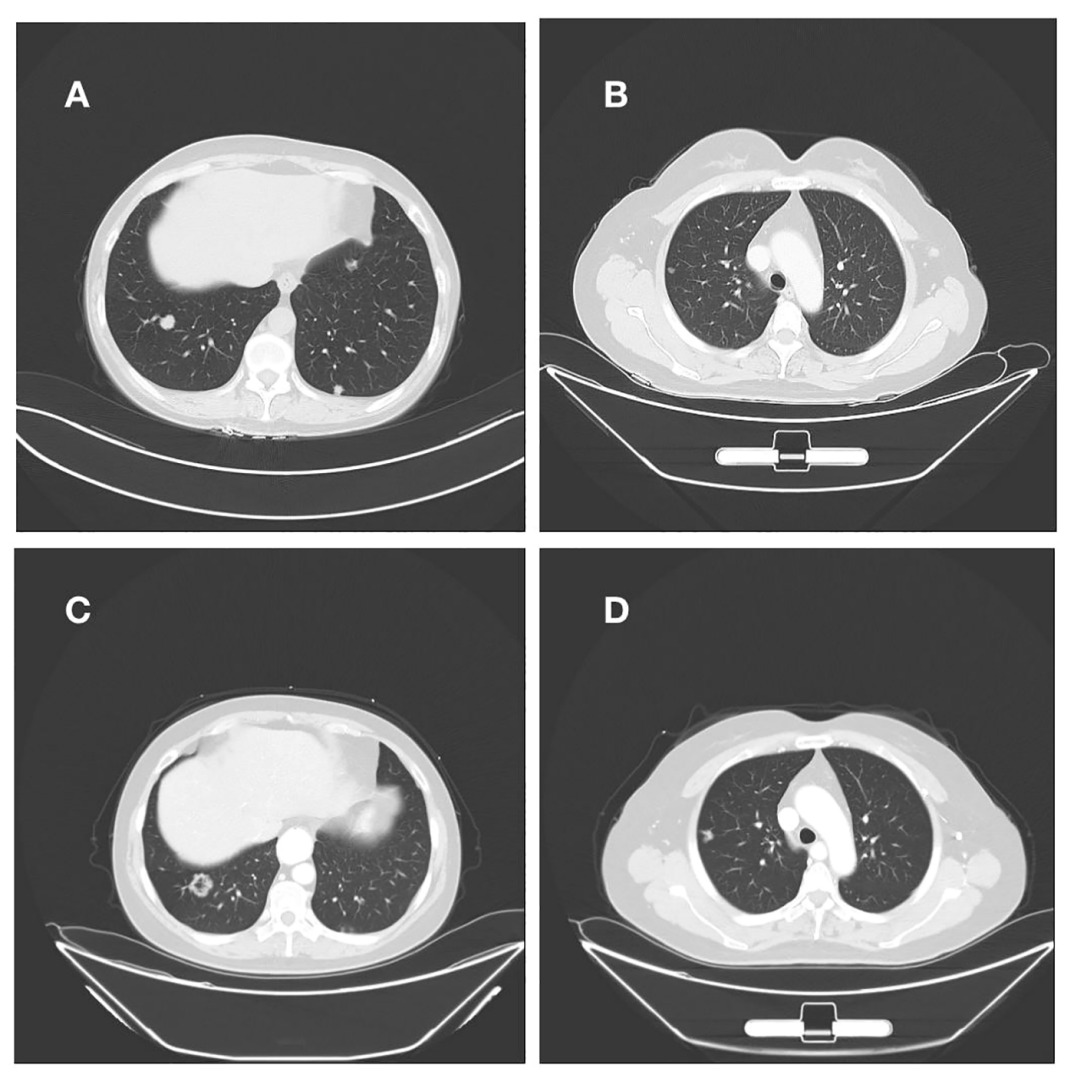
**(A, B)** Right lung nodule on initial admission; **(C, D)** Persistent progression of right lung metastases after multiple cycles of treatment.

The patient had endometrial mucinous adenocarcinoma, and we used IHC to check for the expression of mismatch repair protein and programmed cell death-ligand 1 (PD-L1). This revealed that MLH1 (+, normal), MSH2 (+, normal), MSH6 (+, normal), PMS2 (+, normal), and the tumor tissue PD-L1 expression: Combination positive score(CPS) was 0, in agreement with the findings of the DNA test. IHC and genetic testing both indicated that the DNA mismatch repair system was effective. Immune checkpoint inhibitors are not an option for the patient’s treatment. After then, we modified the therapy to include albumin-bound paclitaxel, carboplatin, and bevacizumab. After four cycles of the aforementioned regimen, repeated CT scans revealed a right lung lesion that had advanced and was about 1.7 cm in diameter. Following two cycles of treatment, the efficacy was assessed for progressive disease (PD) and endometrial cancer recurrence, drawing on the treatment of mucinous adenocarcinoma of the ovary. This patient received oxaliplatin 150 mg intravenous chemotherapy combined with anlotinib capsules 10 mg orally, and after two cycles of treatment evaluated with multiple met During three chemotherapy rounds, a second CT scan revealed that both lung tumors had continued to advance and that numerous additional peritoneal and omental metastases had also developed(**Figure 3**). During therapy and the unchecked course of the illness, the patient never contracted Covid-19/Omicron.

## Discussion

16 instances with onset ages ranging from 37 to 83 years have been recorded globally since 2009 when Mikami originally coined SMMN-FGT (1–5). Adnexal cysts or vaginal drainage characterize the majority of cases. SMMN-FGT is unrelated to high-risk HPV infection, and the majority of patients had minor Ca19-9 or Ca125 elevations in their serum tumor markers. Moreover, SMMN-FGT lesions were endogenous, frequently in the upper middle region of the cervical canal, which is difficult to identify by gynecological examination. All of these clinical aspects make early detection and diagnosis more challenging.

Mikami published the first study on SMMN-FGT in 2009, and lesions have been seen in several areas of the female reproductive tract, including the cervix, endometrium, fallopian tubes, and ovaries, as well as the peritoneum (4) and urinary tract (2). And SMMN-FGT generally shows up morphologically as mucinous epithelial metaplasia, mucinous adenocarcinoma, and other alterations. Due to SMMN-FGT’s relatively uncommon start, there are no clear standards for the diagnosis, management, or prognosis of the condition.

Although the histologic cause of SMMN-FGT is unknown, the existing data are more likely to point to a group of illnesses characterized by mucinous epithelial metaplasia of Mullerian duct origin at various places and malignant lesions that move from benign to malignant. Referring to Mikami’s report, the current diagnostic criteria are simultaneous mucinous differentiation of Mullerian duct-derived epithelium in at least two sites or organs (cervix, endometrium, fallopian tubes, ovaries) based on histomorphological results. MUC6 and HIK-1083 demonstrated positive characteristic immunohistochemistry, with HIK-1083 having a stronger characteristic, although there is no commercial reagent for the HIK-1083 antibody.

All individuals with SMMN-FGT have endometrial lesions and showed endometrial mucinous metaplasia and/or mucinous adenocarcinoma, according to earlier reports. Atypical lobular hyperplasia of the uterine cervix has been described by some researchers as a precancerous form of gastric adenocarcinoma of the uterine cervix (6). Most SMMN-FGT patients have cervical lesions and are characterized by a series of changes ranging from mucinous epithelial metaplasia to mucinous adenocarcinoma, including gastric mucinous metaplasia, lobular hyperplasia, atypical lobular hyperplasia, minimally deviated adenocarcinoma, and gastric mucinous adenocarcinoma. About half of the SMMN-FGT patients have lesions in their fallopian tubes and/or ovaries. If the ovaries are affected, the lesions typically manifest as mucinous cystadenoma of the ovary, mucinous carcinoma of the ovary junction, and in a few rare cases, focal mucinous carcinoma. Urethra and mesentery-related isolated cases have also been documented.

Cervical gastric adenocarcinoma, a non-HPV-related cervical cancer, makes up about 10% of non-HPV-related cervical adenocarcinomas and accounts for 20–25% of cervical malignancies. It is more aggressive than other types of cervical cancer, poorly responsive to radiotherapy, and has a poor clinical prognosis (7, 8). TP53 and STK11 are the two most prevalent genetic alterations in cervical gastric adenocarcinomawh which have the distinctive immunohistochemistry of cervical gastric adenocarcinoma (6, 7) and mildly deviated adenocarcinoma: positive for HIK1083 and MUC6 (6, 9, 10). In terms of its clinical characteristics, appearance, immunohistochemistry, and genetic alterations, SMMN-FGT appears to be closely related to gastric adenocarcinoma (11–13). Further research is still needed to fully understand its mechanism.

The disease should be differentiated from the following diseases in diagnosis: 1)cervical adenocarcinoma involving the uterus and ovaries, 2)endometrial common type gastric mucinous adenocarcinoma involving ovaries and cervix, 3)metastatic gastrointestinal tract tumors, which can be differentiated by relevant immunohistochemical indices, such as CK-7, CK-20, COX-2, and PAX-8; 4)primary malignant tumors in multiple sites of the female genital system. The aggressive tumor components of the SMMN-FGT are all early lesions, and most cases had slow clinical progression according to previous reports. And there are lesion-free areas between tumors of different sites. When mucinous chemosis area, junctional mucinous tumor, and mucinous adenocarcinoma as three lesions exist simultaneously, suggesting that the tumor is not metastatic but occurs at the same time.

The oncogene STK11 is linked to the onset of Peutz-Jeghers Syndrome (PJS), an autosomal dominant illness. The incidence of tumors in this group of patients is 10-18 times higher than in the general population, with tumors of the digestive tract being the most common. If it developed in the female genital system, it shows up as ovarian circumferential tubular gonadotropic tumors, mucinous tumors, cervical gastric adenocarcinoma, and microbial adenocarcinoma. Previous investigations found that certain SMMN-FGT patients had PJS, and genetic testing found that almost all of these patients had the STK11 mutation, however not everyone with the STK11 mutation had PJS. The patient we describe had no PJS, but genetic testing revealed an STK11 nonsense mutation. Recent research has demonstrated that STK11 gene mutations are linked to PD-L1 deletion but not to KRAS mutations, and they cause tumors to lack cytotoxic T lymphocyte infiltration and have a poor response to PD-1/PD-L1 inhibitors (14–16).

According to earlier research, the frequency of KRAS gene mutations in endometrioid adenocarcinoma varies between 12 and 26%. KRAS gene mutations are present in approximately 58% of patients with endometrioid adenocarcinoma with mucinous differentiation, but only 8% to 25% of patients with simple endometrioid adenocarcinoma. This suggests that KRAS gene mutations play a significant role in the mucinous phenotype of endometrial cancer (17). STK11 and KRAS gene mutations predicted worse overall survival in cervical cancer and Non-Small-Cell Lung Cancer (18–20). Rare reports of STK11 and KRAS involvement in the pathophysiology of SMMN-FGT highlight the need for additional research into the mechanisms behind this involvement.

Few examples of SMMN-FGT recurrence following treatment have been reported in prior literature. In contrast to previous reports, the patient, in this case had multiple pulmonary metastases 7 months after surgery with STK11, and although the patient is still in our follow-up, it is obvious that the patient’s prognosis is poor. The patient had invasive cervical adenocarcinoma involving the vagina, with positive postoperative pathology suggesting a positive stump.

## Conclusion

The term “SMMN-FGT” refers to a group of conditions that affect various parts of the female reproductive system, including the cervix, endometrium, fallopian tubes, and ovaries. These conditions are characterized by the simultaneous mucinous differentiation of the Mullerian tube-derived epithelium and progressive progression from benign lesions to malignant tumors. Rarely is the condition described, and there is no consensus regarding its occurrence, clinical characteristics, genetic makeup, modes of diagnosis and care, or prognosis. The condition may be closely linked to the emergence of cervical gastric adenocarcinoma, according to the case reports that are now available, but the precise mechanism is still unknown and further research is still needed.

## Data availability statement

The original contributions presented in the study are included in the article/supplementary material. Further inquiries can be directed to the corresponding author.

## Ethics statement

Written informed consent was obtained from the participant/patient(s) for the publication of this case report.

## Author contributions

RW and HY: introduced the idea; formulation of overarching research goals and aims. HY: Acquisition of the financial support for the project leading to this publication. RW, ML, TH, XW and LC collated figures and documents, and participated in writing papers. All authors contributed to the article and approved the submitted version.
